# Indirect Effect of 7-Valent Pneumococcal Conjugate Vaccine on Pneumococcal Carriage in Newborns in Rural Gambia: A Randomised Controlled Trial

**DOI:** 10.1371/journal.pone.0049143

**Published:** 2012-11-21

**Authors:** Uzochukwu Egere, John Townend, Anna Roca, Abiodun Akinsanya, Abdoulie Bojang, David Nsekpong, Brian Greenwood, Richard A. Adegbola, Philip C. Hill

**Affiliations:** 1 Medical Research Council Unit, Banjul, The Gambia; 2 Barcelona Center for International Health Research, Barcelona, Spain; 3 Faculty of Infectious and Tropical Diseases, London School of Hygiene and Tropical Medicine, London, United Kingdom; 4 GlaxoSmithKline Biologicals, Wavre, Belgium; 5 Centre for International Health, School of Medicine, University of Otago, Dunedin, New Zealand; Aeras, United States of America

## Abstract

**Background:**

Gambian infants frequently acquire *Streptococcus pneumoniae* soon after birth. We investigated the indirect effect of 7-valent pneumococcal conjugate vaccine (PCV-7) on pneumococcal acquisition in newborn Gambian babies.

**Methods:**

Twenty-one villages were randomised to receive PCV-7 to all subjects (11 vaccinated villages) or to infants aged 2–30 months (10 control villages). Other control villagers received Meningococcal C conjugate vaccine. From 328 babies born during the trial, nasopharyngeal swabs were collected after birth, then weekly until 8 weeks of age when they received their first dose of PCV-7. Pneumococcal carriage and acquisition rates were compared between the study arms and with a baseline study.

**Results:**

57.4% of 2245 swabs were positive for *S. pneumoniae*. Overall carriage was similar in both arms. In vaccinated villages fewer infants carried pneumococci of vaccine serotypes (VT) (16.9% [31/184] vs. 37.5% [54/144], p<0.001) and more carried pneumococci of non-vaccine serotypes (NVT) (80.9% [149/184] vs. 75.7% [109/144], p = 0.246). Infants from vaccinated villages had a significantly lower acquisition rate of VT (HR 0.39 [0.26–0.58], p<0.001) and increased acquisition of NVT (HR 1.16 [0.87–1.56], p = 0.312). VT carriage (51.6% vs. 37.5%, p = 031 in control and 46.1% vs. 16.8%, p<0.001 in vaccinated villages) and acquisition rates (HR 0.68 [0.50–0.92], p = 0.013 in control villages and HR 0.31 [0.19–0.50], p<.001 in vaccinated villages) were significantly lower in both study arms than in the baseline study. NVT carriage (63.2% vs. 75.7%, p = 0.037 in control and 67.2% vs. 75.3%, p = 0.005 in vaccinated villages) and acquisition rates (HR 1.48 [1.06–2.06], p = 0.022) and (HR 1.52 [1.11–2.10], p = 0.010 respectively) were significantly higher.

**Conclusion:**

PCV-7 significantly reduced carriage of VT pneumococci in unvaccinated infants. This indirect effect likely originated from both the child and adult vaccinated populations. Increased carriage of NVT pneumococci needs ongoing monitoring.

**Trial Registration:**

ISRCTN Register 51695599

## Introduction

Routine vaccination with a seven valent pneumococcal conjugate vaccine (PCV-7) has reduced invasive pneumococcal disease in vaccinated children and in their unvaccinated adult and infant contacts in many communities [1], [2], [3], [4]. Protection of unvaccinated contacts has been attributed to reduction in carriage of vaccine type (VT) pneumococci in vaccinated children, resulting in decreased transmission of these bacteria [5], [6]. In the Gambia, pneumococcal carriage rates are high and colonisation commences very soon after birth, before the recommended age for administration of the first dose of pneumococcal conjugate vaccine [7]. Any additional indirect, or herd, protection of unvaccinated babies would potentially be of major public health benefit.

Clinical trials of pneumococcal conjugate vaccines among children in South Africa [8] and The Gambia [9] have shown efficacy against invasive pneumococcal disease comparable to that demonstrated in the US [6], [10]. In addition, carriage of pneumococcal serotypes included in the vaccine was reduced in the vaccinees [11]. A cluster Randomized Clinical Trial (RCT) has now documented a reduction in nasopharyngeal carriage of VT pneumococci and a non-significant increase in the prevalence of pneumococci of non-vaccine type (NVT) among unvaccinated subjects 30 months of age and older [12]. To evaluate the indirect effect of pneumococcal vaccination in infants prior to the age of vaccination, a longitudinal carriage study was conducted, as part of this trial, in newborns during their first 8 weeks of life.

## Methods

### Study population

Twenty-one villages in Sibanor, Western Region, The Gambia, were selected as being representative of rural areas in The Gambia, as described previously [13].

### Study design

This single blind, cluster randomized (by village) trial was approved by the joint MRC/Gambia Government Ethics Committee. The conduct of the trial was guided by a Data Safety and Monitoring Board. The protocol for this trial and supporting CONSORT checklist are available as supporting information; see Checklist S1 and Protocol S1. Written informed consent was obtained from the leaders of the communities and from individual participants. The 21 villages were randomly assigned to one of two groups. All participants above the age of 30 months in vaccine group villages received 1 dose of PCV-7 (Prevenar)(Wyeth Lederle Pediatric Vaccines) whilst individuals in this age group resident in the control villages received 1 dose of meningococcal polysaccharide C conjugate vaccine. Younger children in both groups received PCV-7 (1–3 doses according to age group at first dose). Children born during the trial received 3 doses of PCV-7 starting at 2 months of age. This trial was conducted according to the principles of International Conference on Harmonization - Good Clinical Practice guidelines. Details of the randomisation and other methods employed in the trial have been previously described [12].

The vaccinated and control villages were similar at the start of the trial with respect to their basic characteristics, although the Mandinka ethnic group was over-represented in vaccinated communities [12]. All infants born in the study villages between July 2006 and May 2008 were eligible to participate in this ancillary study. The primary outcome measure in this study was carriage of *S. pneumoniae*, considered for the primary analysis in two groups: vaccine type and non-vaccine type pneumococci. A nasopharyngeal swab was collected as soon as possible after delivery, and then weekly until 8 weeks of age when infants were vaccinated with PCV-7 according to the study protocol. Commencement of pneumococcal vaccination from this age was the basis for the 8 week sampling period. The trial was completed prior to introduction of routine PCV-7 into the national immunisation program.

A baseline cross-sectional survey of pneumococcal carriage undertaken two years prior to the trial showed a high prevalence of pneumococcal carriage that was similar in vaccinated and control villages [13]. In another subsequent baseline study, two hundred and thirty-six newborn infants were enrolled in a longitudinal study of pneumococcal acquisition and had a nasopharyngeal swab (NPS) collected at birth, 2 weekly till 6 months of age and then monthly till the end of their first year of life [14]. Carriage data from the baseline (pre-vaccination) study in infants provided a comparison with the carriage data in the ancillary study of the randomised trial.

### Sample size determination

A previous pre-vaccination baseline survey showed that the prevalence of carriage of pneumococci of vaccine type was approximately 40% in infants up to the age of 2.5 months and approximately 30% in those aged 30 months or more [14]. The coefficient of variation between villages was 0.3. Assuming that the study would recruit at least 200 newborns, we calculated that we would have over 90% power, with a type1 error of 5%, to detect a 50% reduction in pneumococcal carriage of vaccine type.

### Sampling process

Nasopharyngeal samples were collected using calcium alginate-tipped swabs (Fisher Scientific, USA). Swabs were inoculated immediately into skim milk tryptone glucose glycerol (STGG) transport medium, placed in a cold box and transferred to the MRC laboratories in Fajara within 8 hours of collection in accordance with the World Health Organisation standard protocol [15]. Inoculated vials were stored at −70°C until they were tested in batches.

### Laboratory analysis

Frozen STGG vials containing swabs were thawed at room temperature and then vortexed for a minimum of 20 seconds. 10 µL aliquot of thawed STGG media was inoculated onto a specific selective medium (gentamicin blood agar). Pneumococcal isolation and serotyping were carried out using standard methods as described previously [16].

### Data management and statistical analysis

All data were double entered into a Microsoft access database and checked for errors. Vaccine serotypes were defined as those contained in PCV-7 (4, 6B, 9V, 14, 18C, 19F and 23F) and serotype 6A. Non-vaccine serotypes were defined as all other serotypes, including non-typeable pneumococci [13], [14]. Acquisition was defined as isolation of *Streptococcus pneumoniae* for the first time. Differences in carriage prevalence of vaccine groups and individual pneumococcal serotypes were tested using generalized linear models of the binomial family with an identity link to estimate risk differences, using a clustered robust variance estimator to adjust for multiple samples per child. Time to first acquisition was defined as the midpoint between a sample without the serotype and the first sample with the serotype, or the midpoint between birth and the first sample date if the first sample was positive. Kaplan-Meier failure functions were used to study time to first acquisition of pneumococci. Mean survival times were calculated by extending the tail of the survival curves to reach the x-axis assuming an exponential distribution if the longest follow-up time was a censored observation. This allowed estimation of mean survival times for acquisition of VT, NVT and any pneumococcus, which in some cases were beyond the end of the study period. A similar approach was used by Cheung et al [11]. It was not possible to calculate median survival times in some cases because too few children had acquired some serotypes before the end of the study. Hazards for acquisition in the trial and in the baseline pre- vaccination study were compared using Cox regression with robust standard errors to adjust for village clustering after checking that the proportional hazards assumption was satisfied. A p-value of <0.05 was considered statistically significant. All statistical analyses were carried out using Stata version 11 (Stata Corporation, TX, USA).

## Results

### Characteristics of the study population

**Figure 1:**
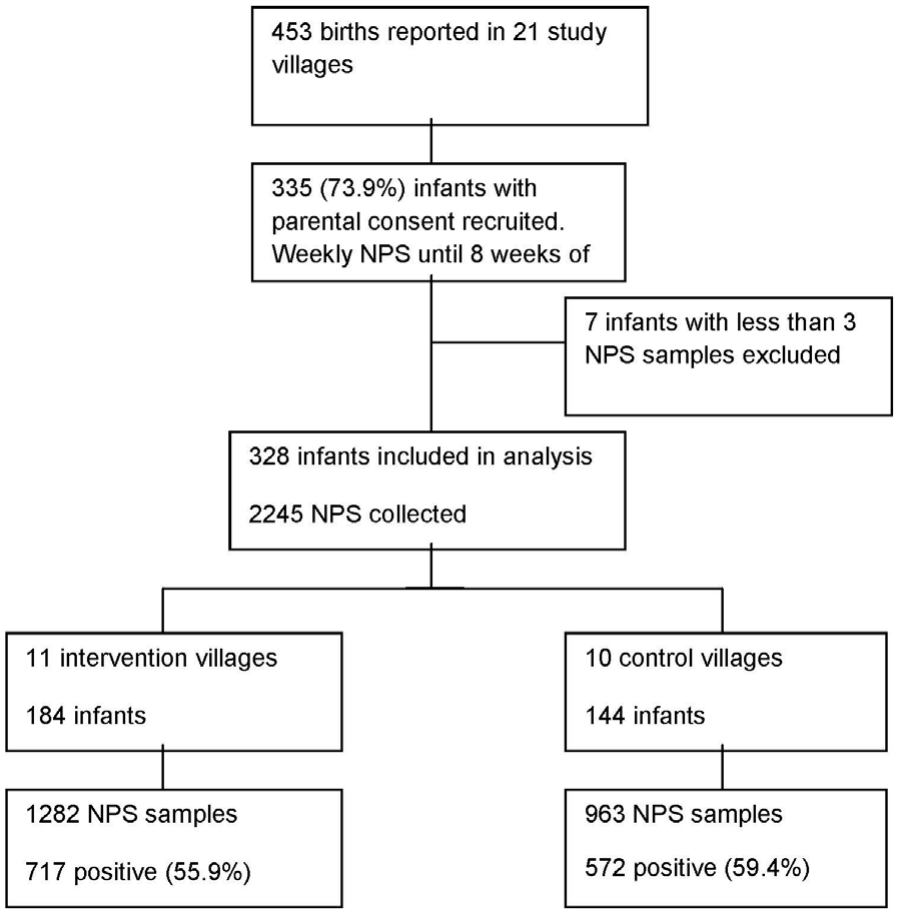
Summary of recruitment, nasopharyngeal samples collected and pneumococci isolated from infants in 21 study villages.

Three hundred and fifty-three new births were reported during the study period. Parental consent was obtained for inclusion of 335 (94.9%) babies in the study. Seven infants who provided fewer than 3 nasopharyngeal samples were excluded, leaving 328 infants (Figure 1). The basic characteristics of infants in the two arms of the study were similar (Table 1). Similar proportions of infants in either arm were born during rainy and dry seasons and within the first or second year following community vaccination with PCV-7. No significant differences were found in administration of antibiotics or reported infections between the two groups.

**Table 1:** Characteristics of infants in the study by village group.

Characteristic	Vaccinated villages	Control Villages
Total number of infants recruited	184	144
Males	100 (54.4)	73 (50.7)
Birth weight, mean (SD) kg	3.4 (.51)	3.5 (.56)
Time of birth		
Rainy season	61 (33.2)	45 (31.3)
Dry season	123 (66.8)	99 (68.7)
In 1st year of community vaccination	89 (48.4)	66 (45.8)
In 2^nd^ year of community vaccination	95 (51.6)	78 (54.2)
Pre-vac pneumococcal carriage (%)+		
VT	46.1	51.6
NVT	67.2	63.2
Overall	93.0	91.8
Exclusive breastfeeding	174 (94.5)	131 (90.9)
Ethnic group		
Jola	102 (55.4)	130 (90.3)
Fula	6 (3.2)	10 (6.9)
Mandinka	68 (36.9)	1 (0.7)
Others*	8 (4.4)	3 (2.1)
Antibiotics given in previous week	15 (8.2)	11 (7.6)
Ear discharge in previous week	6 (3.3)	7 (4.9)
Chest infection in previous week	19 (8.3)	8 (5.6)
Travelled at anytime during the study	50 (27.2)	39 (27.1)

Note:

* includes Serere, Manjago and Sarahule groups present in very small numbers in the villages where the study took place.

+ refers to carriage at any time in infants up to 8 weeks of age. Numbers in parenthesis are percentages except as specified for birth weight.

### Overall pneumococcal carriage by sample and by individual

A total of 2245 NPS were collected, representing a mean of 6.9 samples per infant (median, 7; range 6–8). Nine hundred and sixty-three (42.9%) samples were obtained from infants in control villages while 1282 (57.1%) were from infants in vaccinated villages (Figure 1). *S. pneumoniae* was isolated from 59.4% of samples from infants in the control villages and from 55.9% of samples from infants in the vaccinated villages (p = 0.1 for the difference in proportions). Altogether, 1368 pneumococci were isolated including pneumococci of 63 different serotypes.

Table 2 shows the overall prevalence in the study villages of carriage of individual VT pneumococci and of the 11 commonest NVT pneumococci identified plus non-typeable (NT) pneumococci. This group of 11 NVT serotypes and the NT made up nearly half (47%) of all isolates recovered in the study. Carriage of vaccine serotypes was lower in infants from vaccinated than in those from control villages. This was statistically significant for VT overall (6.0% vs. 20.0%, risk difference −14.0%; p<0.001) and for individual VT 19F and 23F. Although overall carriage of any NVT was significantly higher in infants from vaccinated compared to those from control villages (50.5% vs. 40.5%, risk difference 9.8%; p = 0.010), carriage of the individual NVTs was not significantly different between study arms. As expected, serotypes 1 and 5 were isolated rarely.

**Table 2:** Carriage of selected pneumococcal serotypes isolated at any time during the first 8 weeks of life stratified by study arm.

	Number of samples with carriage (%)					
Serotype	Control		Vaccinated		Risk difference	P
	(n = 963)		(n = 1282)		%	
Vaccine types						
19F	61	(6.3%)	23	(1.8%)	−4.5%	0.015
6A	40	(4.2%)	27	(2.1%)	−2.0%	0.131
23F	54	(5.6%)	8	(0.6%)	−5.0%	0.005
14	16	(1.7%)	6	(0.5%)	−1.2%	0.272
6B	10	(1.0%)	10	(0.8%)	−0.3%	0.715
18C	3	(0.3%)	0	(0.0%)	−0.3%	0.316
9V	4	(0.4%)	3	(0.2%)	−0.2%	0.608
4	7	(0.7%)	0	(0.0%)	−0.7%	0.071
Non-vaccine types						
13	34	(3.5%)	55	(4.3%)	0.8%	0.650
11	32	(3.3%)	55	(4.3%)	1.0%	0.560
10A	24	(2.5%)	53	(4.1%)	1.6%	0.282
16F	27	(2.8%)	49	(3.8%)	1.0%	0.472
15B	18	(1.9%)	56	(4.4%)	2.5%	0.098
NT	26	(2.7%)	39	(3.0%)	0.3%	0.722
19A	17	(1.8%)	33	(2.6%)	0.8%	0.528
34	17	(1.8%)	29	(2.3%)	0.5%	0.688
7F	12	(1.2%)	34	(2.7%)	1.4%	0.241
19B	13	(1.3%)	26	(2.0%)	0.7%	0.545
12	12	(1.2%)	27	(2.1%)	0.9%	0.407
3	17	(1.8%)	22	(1.7%)	0.0%	0.948
Any VT	193	(20.0%)	77	(6.0%)	−14.0%	<0.001
Any NVT	390	(40.5%)	645	(50.3%)	9.8%	0.010
Any pneumococcus	572	(59.4%)	717	(55.9%)	−3.5%	0.325

Note: Figures for vaccine serotypes, NT and the 11 most frequently isolated non-vaccine serotypes are shown.

**Figure 2:**
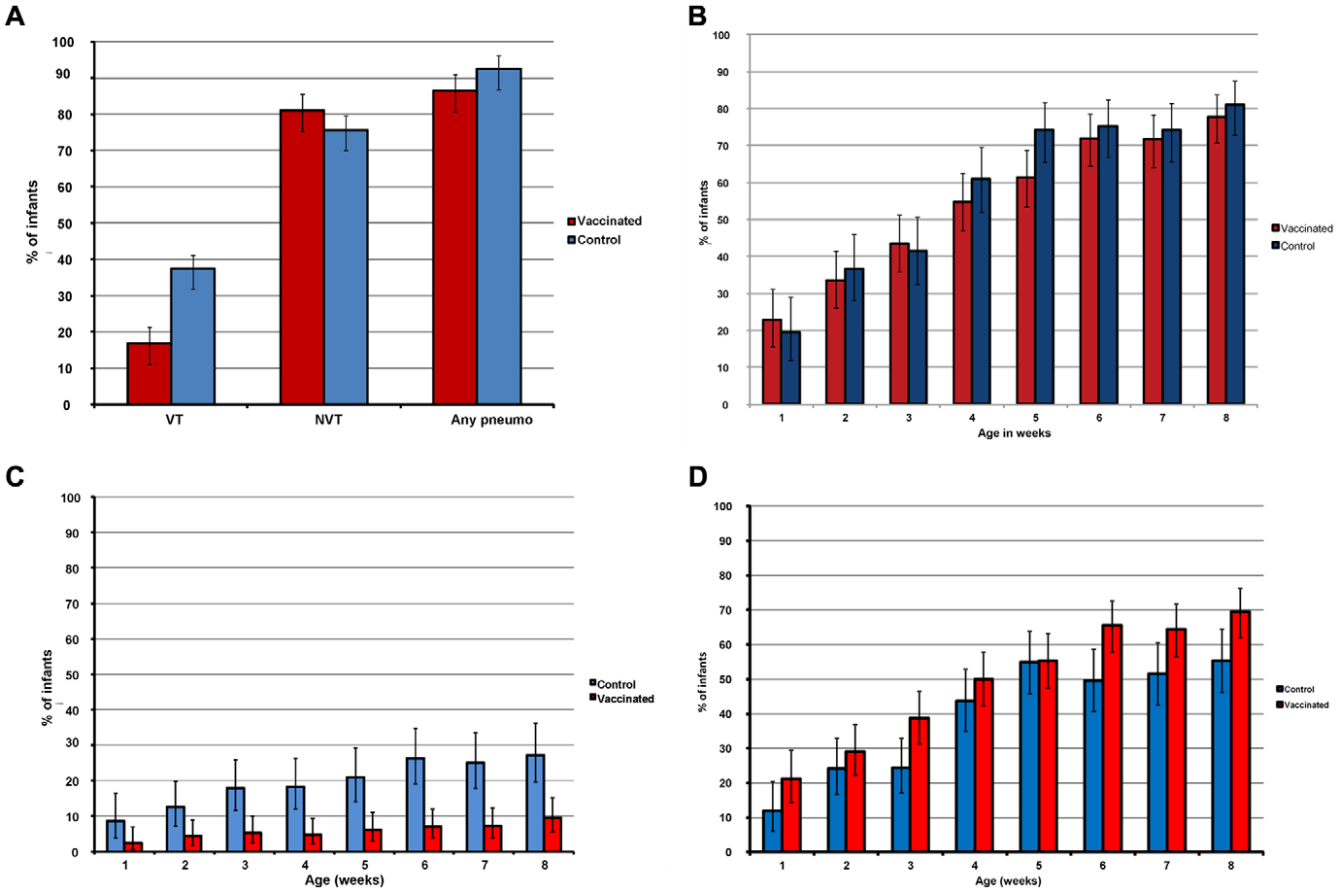
A. Proportion of infants who carried S. pneumoniae at any time in first 8 weeks of life. B. Point prevalence of pneumococcal carriage overall in infants by age and study arm. C. Point prevalence of vaccine serotype carriage by age and study arm. D. Point prevalence of non vaccine serotype carriage by age and study arm.

Ninety-two percent and 86% respectively of infants from control and vaccinated villages born during the study carried pneumococci at some time-point (Figure 2A). Significantly fewer infants from vaccinated compared to control villages carried VT pneumococci (16.9% [31/184] vs. 37.5% [54/144], p<0.001). The proportion of infants who carried NVT pneumococci was higher in young infants from vaccinated villages compared to those from control villages (80.9% [149/184] vs. 75.7% [109/144]) but this difference was not statistically significant (p = 0.246). Compared to the baseline study, VT carriage in infants was significantly lower after vaccination than in the baseline survey in both study arms but the reduction was more pronounced in vaccinated villages (51.6% vs. 37.5%, p = 0.031 in control villages, 46.1% vs. 16.8%, p<0.001 in vaccinated villages). Carriage of NVT in infants was significantly higher after vaccination compared to baseline in both study arms (63.2% vs. 75.7%, p = 0.037 in control villages, 67.2% vs. 75.3%, p = 0.005 in vaccinated villages).

### Weekly point prevalence of carriage

The point prevalence of overall pneumococcal carriage was not different in either study arm except during the 5^th^ week of life when overall carriage was significantly higher among infants from control compared with vaccinated villages (p = 0.022)(Figure 2B). The percentage of infants who carried VT pneumococci was consistently lower in vaccinated villages throughout the first 8 weeks of life. This difference was statistically significant from the 3^rd^ to 8^th^ week of life (Figure 2C). The prevalence of NVT carriage was consistently higher in infants from vaccinated villages compared to those from control villages throughout the first 8 weeks of life (Figure 2D), reaching statistical significance at week 3 (38.7%[65/168] vs. 24.4%[30/123](p = 0.010), week 6 (65.5% [112/171] vs. 49.6%[64/129] (p = 0.006) and week 8 (69.5% [116/167] vs. 55.4 [67/121] (p = 0.014).

### Pneumococcal carriage and year of birth

The proportions of samples with carriage in children born in the first or second year after the start of vaccination were compared to see if there was any cumulative effect from introduction of PCV-7 vaccination. There was no significant difference in total VT carriage in year 1 vs. year 2 and there were similar differences between vaccinated and control arms in the two periods (year 1: 5.3% vs. 25.5%, p<0.001; year 2: 6.6 vs. 15.7%, p = 0.010). The effects of vaccination were also similar in year 1 and 2 on carriage of individual VT. Overall carriage of NVT increased in both study arms in year 1 vs. year 2 but this was not significant (control: 35.5% vs. 44.5%, p = 0.111; vaccinated: 48.2% vs. 52.2%, p = 0.421). Non-vaccine serotypes 7F, 13 and 19A were significantly more common in the second year in both study arms (p = 0.018, p = 0.016 and p = 0.009 respectively). The majority of the 50 serotype 19A isolates (32/44) were found in the second year in intervention villages.

### Time to first carriage and risk of *S. pneumoniae* acquisition

**Figure 3:**
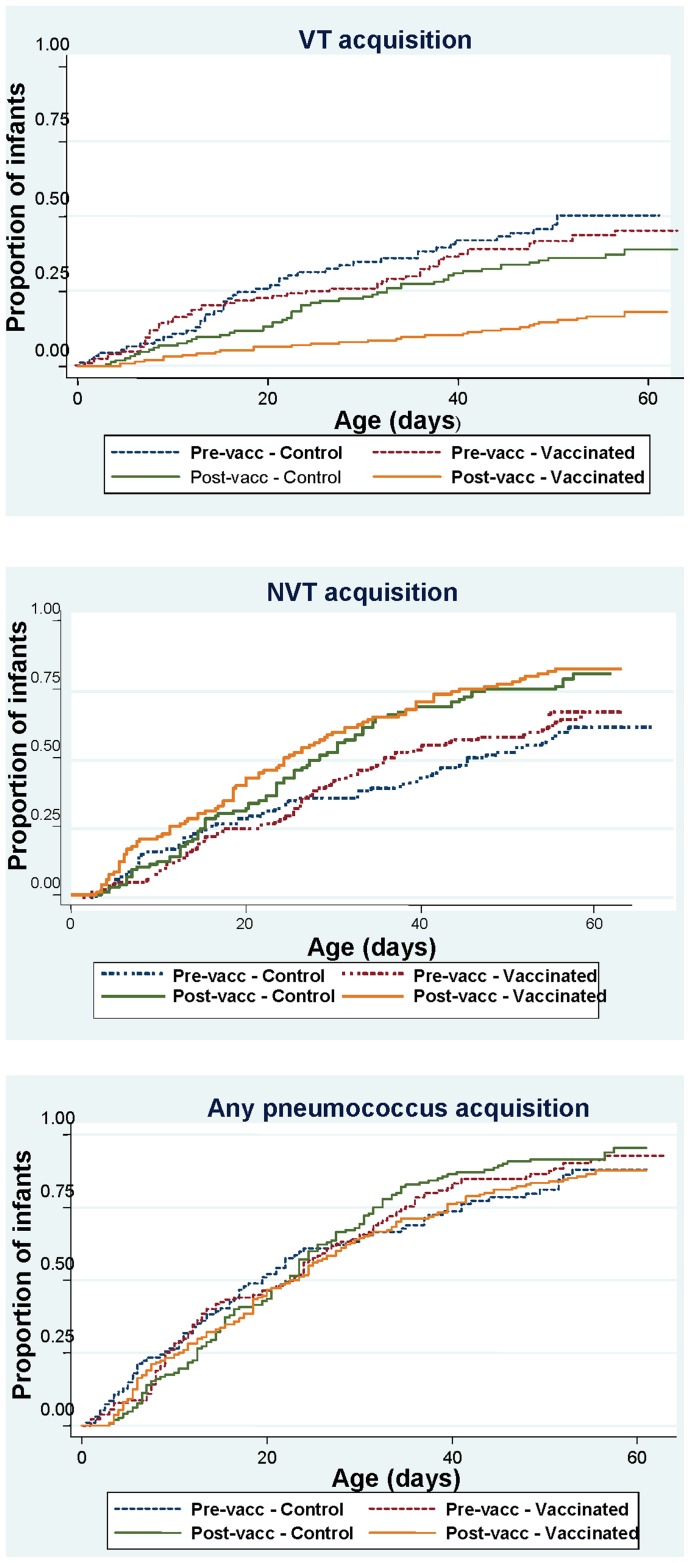
Kaplan-Meier survival curves for time to first acquisition of different serotype groups in the pre-vaccination and post-vaccination surveys by study arm.

We compared pneumococcal acquisition between the study arms and with the baseline survey (Table 3 & figure 3). The hazards for acquisition of any pneumococcus were not significantly different between the study arms. Infants in vaccinated villages had a significantly lower hazard for acquisition of VT (HR 0.39 [0.26–0.58], p<0.001) and VT serotype 19F (HR 0.39 [0.20–0.79], p = 0.009) compared to infants in control villages in the post vaccination study. There was also a higher hazard for acquisition of NVT and NVT serotypes 19A and 11 but these were not statistically significant. Compared to the baseline study findings, there was a significantly reduced hazard of acquisition of VT in infants from both vaccinated (HR 0.31 [0.19–0.50], p<0.001) and control villages (HR 0.68 [0.50–0.92, p = 0.013) in the postvaccination study. Concomitantly the hazard for NVT acquisition was significantly higher in both study arms compared to pre-vaccination study (HR 1.48 [1.06–2.060, p = 0.022 in control villages, HR 1.52 [1.11–2.10], p = 0.010 in vaccinated villages).

**Table 3:** Hazard ratios for first acquisition of selected serotypes and serotype groups in infants from vaccinated and control villages in the main (post-vaccination) study. Comparisons of each arm with the earlier baseline study are also included.

Serotype	post-vaccination				post-vaccination vs. pre-vaccination							
	vaccinated vs. control				control villages				vaccinated villages			
	HR (95% CI)			p-value	HR (95% CI)			p-value	HR (95% CI)			p-value
Vaccine types	0.39	(0.26–	0.58)	<0.001	0.68	(0.50–	0.92)	0.013	0.31	(0.19–	0.50)	<0.001
Non-vaccine types	1.16	(0.87–	1.56)	0.312	1.48	(1.06–	2.06)	0.022	1.52	(1.11–	2.10)	0.010
Any pneumococcus	0.83	(0.59–	1.17)	0.298	1.14	(0.85–	1.52)	0.394	0.87	(0.61–	1.26)	0.474
19F	0.39	(0.20–	0.79)	0.009	1.80	(0.64–	5.04)	0.261	0.55	(0.34–	0.89)	0.016
23F	0.29	(0.07–	1.21)	0.088	0.63	(0.33–	1.18)	0.150	0.42	(0.12–	1.50)	0.179
6A	0.64	(0.30–	1.39)	0.264	1.46	(0.58–	3.68)	0.421	0.78	(0.27–	2.21)	0.634
19A	1.67	(0.54–	5.14)	0.372	1.04	(0.25–	4.26)	0.958	3.70	(0.83–	16.40)	0.085
7F	1.01	(0.28–	3.68)	0.985	2.21	(0.32–	15.27)	0.421	>1	-	<0.001*	
13	0.97	(0.40–	2.34)	0.942	3.82	(0.91–	15.98)	0.067	3.55	(1.69–	7.44)	0.001
11	1.31	(0.65–	2.63)	0.447	2.10	(0.54–	8.08)	0.283	3.91	(0.85–	18.07)	0.080

* 9 children in the vaccinated villages had serotype 7F isolates, all of these were in the post-vaccination survey. Hazard ratios comparing the hazards for first acquisition of pneumococci with those found in the pre-vaccination survey in each arm of the trial are also shown.

The mean times to first acquisition of any pneumococcus were 29.5 days and 32.8 days in infants from the vaccinated and control villages respectively (Figure 3). The mean time to acquisition for VT pneumococci was 120 days in infants from control villages and 304 days in those from vaccinated villages. Mean times to first acquisition of NVT pneumococci were 37.7 days and. 44 days in infants from vaccinated and control villages respectively.

No statistically significant associations were found between breastfeeding, ethnicity, gender, household size or number of children <5years in the household and time to first acquisition of a pneumococcus.

## Discussion

To the best of our knowledge, this is the first report of indirect protection of newborn infants against PCV-7 serotypes from Africa. We found significantly reduced carriage and risk of first acquisition of VT pneumococci within the first 8 weeks of life in infants born into communities where the whole population had been vaccinated with PCV-7 compared to those born in control communities where only those <30 months had been vaccinated. In parallel, overall carriage rate and risk of acquisition of non-vaccine serotypes (NVT) were increased in these infants compared to controls. A significant reduction in carriage and acquisition rate of VT was also demonstrated in both groups of villages when compared to findings in the previous baseline cohort study.

Protection against VT carriage has been demonstrated previously among American Indian infants living in PCV7-vaccinated communities compared to their counterparts living in control communities and this was attributed to reduced transmission of VT from vaccinated children [17]. In addition, the proportion of isolates that were NVT was significantly higher in infants in vaccinated communities compared to controls. Indirect protection against VT carriage has also been demonstrated among children and adults living with a PCV-7-vaccinated child [18]. The findings from these studies among American Indian children are similar to ours. The epidemiological characteristics of pneumococcal disease in the American Indian population is known to be similar to that seen in The Gambia, with high rates of carriage and invasive disease [19]. However, our study has shown indirect protection of infants at a much younger age than in these studies and for the first time in a developing country setting.

Since pneumococcal transmission in the community is probably driven mainly by young children [20], as has also been shown previously in the Gambia [21], we expected to find little difference in carriage among the unvaccinated newborns. The absolute drop in VT carriage from baseline was about twice as much in infants from villages where the whole population had received PCV-7 than in infants from control villages. It is possible that a proportion of newborn infants in rural Gambia first acquire pneumococci from their mothers and that maternally acquired antibodies against VT (all mothers in vaccinated villages received one dose of PCV-7) could have played a role in the reduced VT carriage and acquisition rates seen in infants in vaccinated villages. Enhanced concentrations of IgG to specific pneumococcal serotypes have been demonstrated in Bangladesh [22] and in Papua New Guinea [23] among infants born to mothers who received pneumococcal polysaccharide vaccine in late pregnancy. A three-fold increase in anti-pneumococcal IgG in the colostrum was also demonstrated previously in Gambian mothers who received a pneumococcal polysaccharide vaccine in pregnancy [24]. A similar study in the USA demonstrated enhanced IgG concentrations in infants of vaccinated pregnant women who received pneumococcal polysaccharide vaccine and lower nasal carriage of pneumococcal serotypes in the infants up to the age of 16 months [25].

In our study the proportion of isolates that were NVT was significantly higher in infants in vaccinated communities compared to those from control. Furthermore, though the risk of NVT acquisition was not significantly different between both study arms, it was significantly higher in each of the study arms when compared with findings from the baseline cohort study. Replacement carriage with NVT has been demonstrated in randomised controlled trials [26], [27], [28], [29] and observational studies [30], [31], [32]. This trend in newborns in The Gambia is of concern given the increased frequency of NVT invasive disease which has been observed in some, but not all, populations where PCV-7 is routinely used [33], [34]. We noted no overall difference in the prevalence of VT or NVT serotypes by year since the start of the study. However the individual NVT serotypes 7F, 13 and 19A were all significantly more common in second year following vaccination, and the majority of the serotype 19A isolates were found only in second year in intervention villages. Serotype 19A has become the most frequently carried serotype and the leading cause of invasive disease and respiratory infections in the United States after the introduction of PCV7 [35], [36]. We may have observed a true delayed replacement effect of 19A and this requires careful monitoring.

This study had some limitations. A follow up period of just 8 weeks, necessitated by the vaccination with PCV-7 of all infants in the study at this time, weakened the ability of the study to demonstrate whether the observed indirect effects would persist, disappear or even increase over time. The duration of carriage could not be assessed as most carriage losses were censored. We have used data from a longitudinal carriage study among infants born in the study communities nearly 2 years prior to the start of vaccination to make inferences about the extent of the indirect protection of newborns in the control villages. Though pneumococcal carriage rates have remained the same in Gambia in the years preceding PCV vaccination, temporal trends in the study villages could have reduced comparability between these data. However, whatever changes that might have occurred should have been distributed equally among the study arms because of randomisation and not affected the main conclusions of the study. The possible role of maternally acquired anti-pneumococcal antibodies in reducing carriage in infants in vaccinated villages could only be speculated on as this was not studied. The study was designed to detect differences in carriage of any VT or any NVT pneumococci. We conducted further analyses of individual serotypes. The chance of finding spurious effects is increased in these secondary analyses, due to multiple comparisons, and consequently these findings for individual serotypes need to be interpreted with caution.

In summary, this randomised controlled trial in rural Gambia has demonstrated that PCV-7 vaccination results in strong indirect protection against pneumococcal carriage of vaccine serotypes during the first 8 weeks of life. Comparison of the differences in pneumococcal carriage between communities where the whole population was vaccinated, communities where only under 30 month old children were vaccinated and the same villages prevaccination suggest that this strong indirect effect comes from both under 30 month and over 30 month old vaccinated populations. While evidence of replacement carriage with NVT necessitates the need for continued surveillance, the combined direct and indirect effects of PCV-7 enhance the recommendation for widespread implementation of PCVs into routine vaccination schedules in countries where they are needed most.

## Supporting Information

Protocol S1 **Trial Protocol.**
(PDF)

Ethical Approval S1 **Trial Ethical Approval.**
(PDF)

Checklist S1 **CONSORT checklist.**
(DOC)
