# Supplementary material for: Indirect Effect of 7-Valent Pneumococcal Conjugate Vaccine on Pneumococcal Carriage in Newborns in Rural Gambia: A Randomised Controlled Trial
Source: PLoS One. 2012 Nov 21;7(11):e49143. doi: 10.1371/journal.pone.0049143 (PMC3504064; doi:10.1371/journal.pone.0049143)
Supplement: Ethical Approval S1 — Trial Ethical Approval. (PDF) [file pone.0049143.s002.pdf]

c/o MRC Laboratories Fajara
P. O. Box 273, Banjul
The Gambia, West Africa

The Gambia Government / MRC Laboratories Joint

ETHICS COMMITTEE

Fax: +220 – 4494498 or 4496 513
Tel: +220 – 4494 073-9 ext. 409

27th February 2006

Prof. Richard Adegbola
Head
Bacterial Diseases Programme
MRC, Fajara

Dear Prof. Adegbola

Re: 1032v2 Community randomised controlled trial to assess the impact of vaccination with a pneumococcal conjugate vaccine on nasopharyngeal carriage of pneumococci in the Gambia.

Thank you for resubmitting the above proposal which was reviewed and approved by the Joint Gambia Government/MRC Ethics Committee at its meeting on Friday 24th February 2006.

With best wishes,

Yours sincerely

Mr. Malcolm Clarke
Chairman
Gambia Government/MRC Joint Ethics Committee

Cc:

The Gambia Government / MRC Laboratories Joint Ethics Committee:

Mr Malcolm Clarke, Chairman
Mrs Kathy Hill, Secretary
Mrs Norwoh Ali, 2nd Secretary
Dr Ousman Nyan, Scientific Advisor
Mr Dawda Jagne
Mrs Bertha Mboge
Dr Tumani Corrah
Professor Hilton Whittle
Dr Stephen Howie
Dr Mariatou Jallow
Dr. Lamin Sidibeh
Mr. Malamin Sonko
